# Mildew detection in rice grains based on computer vision and the YOLO convolutional neural network

**DOI:** 10.1002/fsn3.3798

**Published:** 2023-11-06

**Authors:** Ke Sun, Mengdi Tang, Shu Li, Siyuan Tong

**Affiliations:** ^1^ College of Life Sciences Anhui Normal University Wuhu China

**Keywords:** computer vision, microscopic images, moldy rice, quick detection, YOLO convolutional neural network

## Abstract

At present, detection methods for rice microbial indicators are usually based on microbial culture or sensory detection methods, which are time‐consuming or require expertise and thus cannot meet the needs of on‐site rice testing when the rice is taken out of storage or traded. In order to develop a fast and non‐destructive method for detecting rice mildew, in this paper, micro‐computer vision technology is used to collect images of mildewed rice samples from 9 image locations. Then, a YOLO‐V5 convolutional neural network model is used to detect moldy areas of rice, and the mold coverage area is estimated. The relationship between the moldy areas and the total number of bacterial colonies in the image is obtained. The results show that the precision and the recall of the established YOLO‐v5 model in identifying the mildewed areas of rice in the validation set were 82.1% and 86.5%, respectively. Based on the mean mildewed area identified by the YOLO‐v5 model, the precision and recall for light mold detection were 100% and 95.3%, respectively. The proposed method based on micro‐computer vision and the YOLO convolutional neural network can be applied to the rapid detection of mildew in rice taken out of storage or traded.

## Graphical abstract

A YOLO‐v5 model is established to automatically detect moldy regions in MCV images of rice contaminated by mold. Using MCV combined with the YOLO‐v5 model, we can detect the slight moldiness in unhulled rice with a total viable bacterial count of 10e3–10e5 CFU/g. Compared with the traditional method, this method is real‐time and low cost, and can be widely used in rice safety evaluation in the rice market.

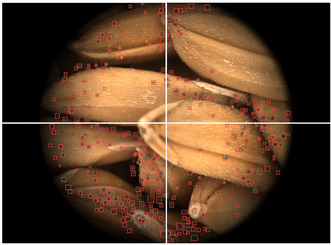

## 1 INTRODUCTION

Rice is one of the main food crops in the world, and a significant part of China's agricultural area is devoted to its production. Its safe production is crucial to ensuring not only China's but also the global food supply. Rice is rich in carbohydrates, proteins, fats, vitamins, and minerals and thus provides a natural culture medium for microorganisms. During the storage and transportation of rice, changes in temperature, humidity, and moisture in the storage environment can lead to the growth and reproduction of dominant molds such as *Aspergillus flavus* and *Aspergillus niger*. This phenomenon not only causes huge economic losses but also poses serious harm to the health of breeding animals and human health (Mannaa et al., 2017; Santos et al., 2022). Due to the enormous amounts of rice produced globally, there is a high demand for microbial indicator detection methods, which are commonly applied when the rice is taken out of storage or traded. At present, the commonly used microbial indicator detection methods mainly include plate culture methods, artificial sensory evaluation methods, and enzyme‐linked immune response methods. However, due to the time‐consuming nature, low accuracy, or high cost of these methods, microbial indicator detection can only be performed on small samples (Gong et al., 2021). Under these circumstances, rice mildew is relatively easy to miss, and thus more convenient, fast, and accurate detection methods are needed, which would facilitate the testing of larger samples.

Non‐destructive testing methods based on optical and volatile gas signals have the advantages of being fast and requiring no preprocessing (Chen et al., 2021; Wen et al., 2022). Using such methods, significant changes in color, odor, morphology, and texture can be observed in normal and severely mildewed cereals (Wan et al., 2019). In recent years, a series of methods have emerged that utilize odor sensors, spectral technology, hyperspectral imaging technology, and computer vision in combination with chemometrics to identify and detect moldy grains. These methods have outstanding real‐time performance and have high application value in grain quality evaluation.

In their work using odor sensors, Shen et al. (2016) used electronic nose technology combined with linear and least square discriminant analysis models to detect cereal samples infected with *A. flavus*, parasitic Aspergillus, and penicillium, with the overall detection rates of the two models reaching 100% and 97.4%, respectively. Jiarpinijnun et al. (2020) achieved early detection of fungal infection in brown rice using electronic nose equipment combined with principal component analysis, partial least squares regression models, and support vector machine models. Wang et al. (2021) used colorimetric sensor technology and a fivefold cross‐validation method to determine the optimal parameters of a qualitative identification model for wheat mildew and the number of principal components (PCs). Using a support vector machine recognition model, they achieved a 100% correct recognition rate for independent wheat samples. However, the detection process involving odor sensors requires long time intervals for volatile molecule adsorption and deionization and is thus not suitable for rapid testing.

In their work using spectroscopy and hyperspectral imaging, Cong et al. (2018) achieved non‐destructive detection of the total number of mold colonies in rice using a model combining gray wolf optimization and support vector regression. Jiang et al. (2018) used an array spectrometer based on principal component analysis to perform cluster analysis on wheat samples infected with different molds and established a linear discriminant analysis model with an accuracy rate of over 90%. Chen et al. (2021) identified 21 characteristic substances from volatile organic compounds in rice samples using gas chromatography with ion migration spectrometry. Combining principal component analysis and *k*‐means clustering, they established a clustering model that can be used to quickly identify the degree of rice mildew. Jia et al. (2022) established a back‐propagation neural network model with an ant colony optimization classification model using a combination of standard normal variables and non‐information variable elimination to process hyperspectral image data of five grades of corn seed mold in the same variety. Liu et al. (2023) proposed a method for identifying sunflower seed mildew grades based on near‐infrared diffuse reflectance and transmission fusion spectroscopy, along with a one‐dimensional convolutional neural network (CNN). However, generic near‐infrared spectrometers can only measure sample points and require the sample to be homogenized, which is not suitable for rapid rice contamination detection. Moreover, multispectral and hyperspectral equipment is expensive, and the image data acquisition times are long.

In computer vision applications, Pan et al. (2017) used computer vision, support vector machines, partial least squares discriminant analysis, and continuous projection algorithms to collect and analyze images of rice samples with different degrees of mildew, achieving an overall accuracy in distinguishing the mildewed parts of over 90%. Chen et al. (2019) used an independently developed machine vision system to simultaneously detect four types of defects in red indica rice: broken grains, chalkiness, breakage, and spots, with recognition accuracy rates of over 93%. Due to the limitations of field size and resolution, traditional computer vision has low sensitivity to mildew detection. Compared with traditional machine learning, deep learning technology has obvious advantages in classification ability and speed.

Sun et al. (2016) compared the effect of traditional machine learning and the LeNet5 CNN on image recognition of mildewed rice, and the results showed that the early LeNet5 deep learning algorithm had a great advantage in recognition speed and accuracy. However, conventional computer vision methods are unable to detect rice samples with mild mildew contamination, and it is difficult to detect mildly mildewed grains with a total viable bacterial count (TVC) of 10^5^–10^6^ CFU/g using methods at conventional observational scales (Sun et al., 2022; Zhou et al., 2008). Sun et al. (2022) used micro‐computer vision (MCV) technology to improve the sensitivity to mildew features and used an advanced YOLO CNN to recognize the location of *A. niger*, *Penicillium orange*, and *Aspergillus griseus* on single rice grains with an accuracy of 89.26%, 91.15%, and 90.19%, respectively. Conventional computer vision methods can directly detect obvious bacterial colonies in mildewed rice images; however, bacterial colonies of mildly mildewed rice are small, and MCV techniques are required for feature extraction (Barsanti et al., 2022; Sun et al., 2022). At present, detection methods for rice mold based on MCV only realize the detection of a single kind of mold on isolated rice grains. Detection methods suitable for multiple grain analysis and multiple mold types have not been presented.

Smaller observation scales also contain more complex image information, and powerful recognition models are required to recognize images acquired using MCV. The YOLO (You Only Look Once) CNN is an advanced deep learning model with strong image analysis and target detection capabilities and can locate multiple targets in images (Redmon et al., 2016). Compared with earlier networks such as LeNet and Alexnet, the YOLO model has a deeper network structure and higher recognition speed than R‐CNN and Fast R‐CNN, and the detection time of targets in images is usually less than 0.1 s. It has been widely used in the fields of face recognition, disease and pest monitoring, transportation, and medical imaging, but its adoption in the field of microbiology is still limited (Cao et al., 2019; Chen et al., 2021; Jiang et al., 2021; Li et al., 2022). The YOLO CNN model is suitable for the recognition of mold in complex microscopic rice images. YOLO‐v5 is the fifth generation of the YOLO model, and its training and recognition speeds and model size are compared with those of its previous generations; thus, it is currently the most commonly used version of YOLO.

In this paper, in order to promote the practical application of MCV in rice mold detection, rice samples were loaded into petri dishes, and images of molded rice were captured using MCV combined with a 9‐point acquisition method. Then, a YOLO‐V5 model was established for detecting mildew areas in images, and its accuracy was verified. Finally, the proportion of mildew areas in each sample image was obtained using this model, and correlation analysis was conducted with the actual total number of colonies to determine the proportion threshold of mildewed areas in mildly mildewed samples. The complete detection method allows the detection of mild rice mildew using MCV and the YOLO model.

## 2 MATERIALS AND METHODS

### 2.1 Simulated storage of rice after inoculation

In this study, we selected a rice variety that is naturally moldy in the field (indica rice, purchased from Huolonggang Town, Wuhu City, Anhui Province, China) as the research object. The steps for mold inoculation and simulated storage are as follows:

1. The rice sample was placed in an oven and baked at 80°C for 4 h to remove the original field mold that resided on the rice grains. The dried rice grains were placed in a set of 60 mm circular culture dishes (10 g of rice in each culture dish).
2. The naturally moldy rice was washed with sterile distilled water to prepare a spore suspension. Through polymerase chain reaction testing, bacteria suspension was detected using in vitro amplification, and the results showed that the mold on the surface of the rice came from *Aspergillus* and *Fusarium*. The spore suspension was inoculated in potato glucose agar. The resulting culture dish was placed in an incubator with constant temperature and humidity (temperature 28°C, relative humidity 90%) for 24–36 h. The concentration in the spore suspension was measured using a plate counting method and then diluted to 1.5 × 10^5^ CFU/mL.
3. 70 Petri dishes containing 10 g of rice grains were created, and 1 mL of spore suspension was inoculated into each culture dish and shaken well to fully impregnate each grain of rice. Then, the dishes were stored under constant temperature and humidity conditions of 28°C and 90% relative humidity for 13 days, and a group of samples (10 petri dishes) was inspected every 48 h to determine when a high degree of mold formation had been reached, i.e. when the mold content per gram of grain was greater than 10^6^ CFU/g.

### 2.2 Microscopic image acquisition of rice samples

**FIGURE 1:**
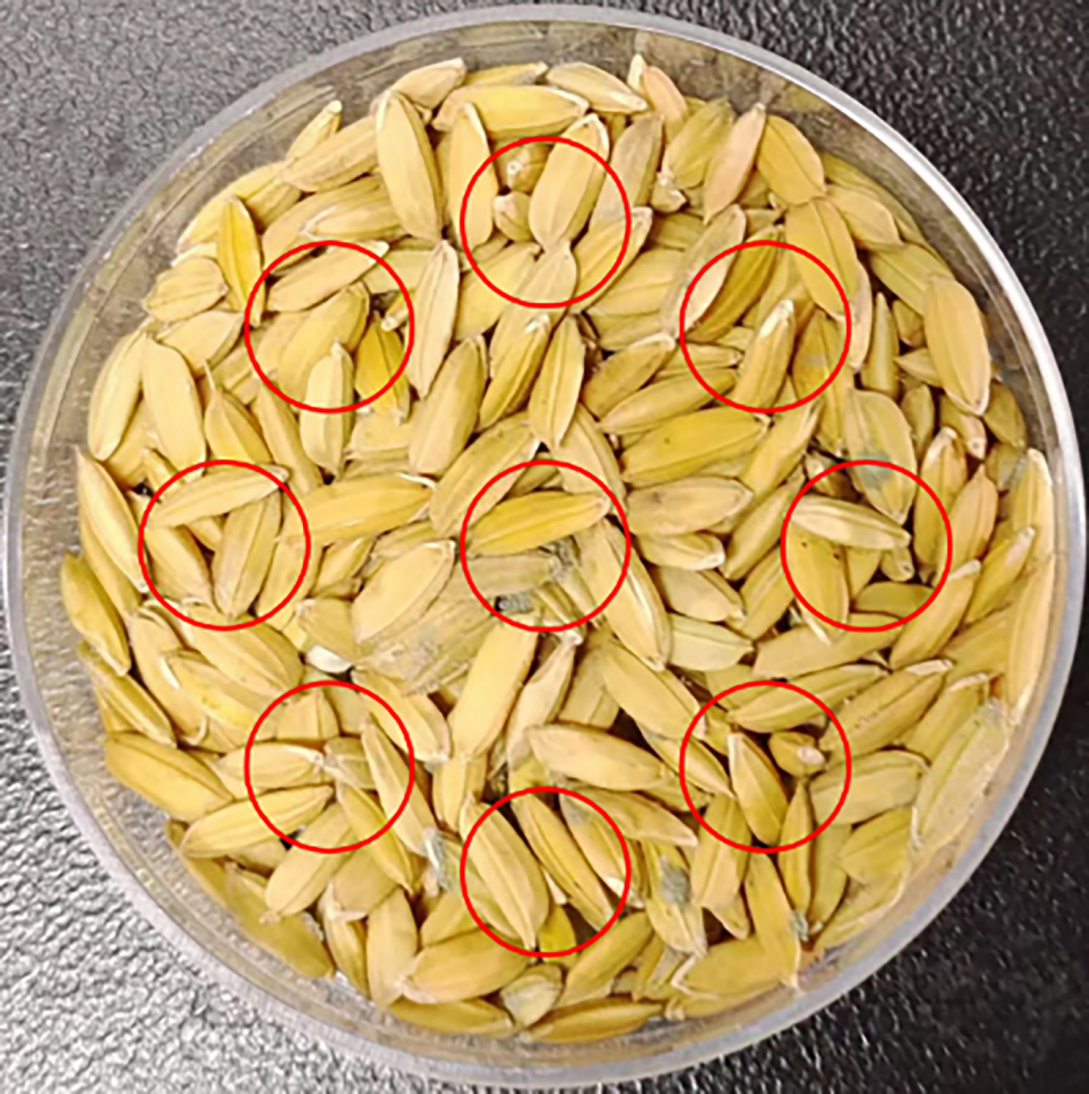
MCV images of unhulled rice (9 imaging regions in the same culture dish).

In this study, the combination of a microscope lens and a Dahua A7A20MU30 color array industrial camera (purchased from Hangzhou Huarui Technology Co., Ltd., China) was used to collect moldy rice MCV images in each culture dish at 9 points. In this process, it is important to ensure that the 9 sample points are evenly distributed and do not overlap each other, so that all details are clearly captured. Therefore, 9 MCV images were obtained for each culture dish (10 g of rice) (as shown in Figure 1), with a captured image size of 2560 × 1876 pixels and a spatial resolution of about 0.02 mm per pixel. During simulated storage, a group of samples (10 petri dishes) were taken out approximately every 48 h for image collection, and 90 images were obtained each time. A total of 630 microscopic images of moldy rice were obtained over 13 days.

### 2.3 Image labeling

**FIGURE 2:**
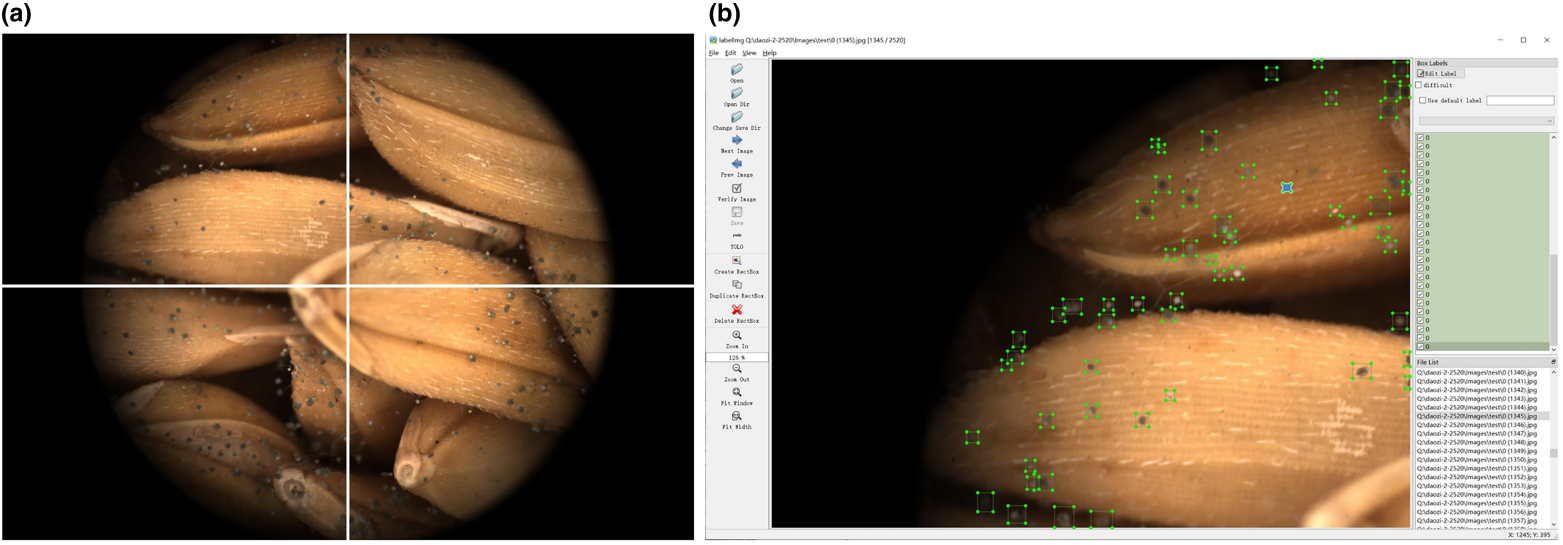
Marking of the mildewed areas (b) in the original MCV image (a) of the rice.

The YOLO CNN is a deep learning algorithm used for target detection and thus requires manual labeling of the target areas. In order to highlight the mildewed (i.e. target) areas in the MCV rice images, each image was divided into four parts, resulting in a resolution of 1280 × 938 pixels for each part. As shown in Figure 2, the Labelimg image marking tool developed in Python was used to mark mildewed areas in all MCV images. In order to extract the mildew areas more accurately, sporangia was selected as the main recognition object, as it has a certain optical structure and area layout in images.

### 2.4 Establishment of the YOLO‐v5 model

The fifth‐generation YOLO model (YOLO‐v5) was used to establish a model for identifying moldy rice areas. The architecture and main functions of the model were consistent with those of Sun et al. (2022), which was used to extract target features from images, then aggregate the target features and build a model for identifying the same targets.

In this study, a total of 38,061 mildewed areas in 2520 microscopic images of rice grains were identified. The rice microscopic images were randomly divided into training sets and test sets at a 6:4 ratio, resulting in 1512 training set images and 1008 test set images. In order to improve training speed and reduce memory consumption, a YOLO‐v5s model was adopted, which is a version of the YOLO‐v5 model with fewer layers and nodes for easier training and deployment. The model was built using Pycharm (JetBrains, Czechs) and the YOLOv5 master toolbox (https://github.com/ultralytics/yolov5). The YOLO‐v5 hyperparameters were as follows: the input image resolution was set to 1280, the batch size was set to 8, the model learning rate was set to 0.01, and the number of epochs was set to 50. Techniques such as Mosaic enhancement and image rotation were also applied.

In order to reduce the probability of the model misjudging the background and improve the fitting degree of the output boundary box and mildewed areas, the confidence threshold of the model was set to 0.5. After the training was completed, the optimal model was determined based on the change in the box loss, the object loss, and the mean average precision at a confidence threshold of 0.5 (mAP_0.5_). Then, to verify the accuracy of the model, the confusion matrix, precision, and recall of the detection results of mildewed areas were obtained.

(1) $$\;\text{Precision}=\frac{\mathrm{tp}}{\mathrm{tp}+\mathrm{fp}}$$

where tp and fp are the numbers of true and false positives, respectively.

(2) $$\;\text{Recall}=\frac{\mathrm{tp}}{\mathrm{tp}+\mathrm{fn}}$$

### 2.5 Analysis of the relationship between the area of mildewed rice areas and the total number of bacterial colonies

In order to obtain a more accurate relationship between the mildewed rice area and the total number of bacterial colonies, it is necessary to repeat experiments on rice inoculation and simulated storage. In this experiment, a group of samples (10 petri dishes) were extracted every 48 h during the simulated storage period of 13 days to determine the TVC. As the current detection target for moldy rice refers to the brown rice portion after hulling, half of the rice was hulled. The purpose of this experiment was to evaluate whether the degree of unhulled rice mildew can be used to estimate the degree of mildew in brown rice in actual production processes by observing the fitting degree of the TVC curve of the two samples. Therefore, the rice particles were roughly divided in each culture dish (containing 10 g of rice) into two parts by mass. After weighing and recording the mass data, they were poured into test tubes. One portion was kept in its unhulled state, while an inspection huller (Rizhao Liang’an Storage Equipment Co., Ltd.) was used for hulling, that is, to obtain a certain amount of brown rice.

Next, 10 mL of sterile distilled water was added to 20 of the test tubes, and the contents were mixed and vibrated using an SK‐1 rotary mixer (Jiangsu Jintan Yitong Electronics Co., Ltd.) to fully elute the mold spores on the unhulled and brown rice grain surfaces, and unhulled and brown rice spore suspensions were obtained. On the first day, after diluting the suspension 10 times, 0.5 mL were extracted and inoculated into potato glucose agar supplemented with chloramphenicol. The resulting 20 Petri dishes (90 mm circular Petri dishes) were placed in a constant temperature and humidity incubator (temperature 28°C, relative humidity 90%) for 36 h, and the total number of unhulled and brown rice colonies was calculated using the colony plate counting method. During simulated storage, the dilution factor was gradually increased by an order of magnitude based on the growth of mold colonies, and a total of 7 sets of measurements were obtained, with each set containing 20 quality measurements and 20 TVC values. Subsequently, the data were processed and analyzed. The equation for calculating the TVC per gram of rice (or brown rice) is shown in (3).

(3) $$\;\mathrm{TVC}=\frac{\text{Total number of colonies in each culture dish}\times \text{Dilution ratio}}{\text{Corresponding grain quality of rice}\;\left(\text{or brown rice}\right)}$$

Then, a correlation analysis was conducted on the changes in the TVC of rice and brown rice and calculated the determination coefficient *R* ^2^.

Finally, the proposed YOLO model was used to identify the mildewed areas in the images. The mean proportion of mildewed area in each image (MAI) was calculated for each grain image. The calculation method is shown in Equation (2):

(4) $$\mathrm{MAI}=\frac{1}{36}\sum_{i=1}^{9}\sum_{k=1}^{4}\frac{M}{S}$$

where *i* is the index of the image location examined; *k* is the index of the 9 captured regions of rice sample; *M* is the area of the mildewed area in one of the 4 divided images; and *S* is the total area of the divided image (1280 × 938). Then, regression analysis was performed on the TVC and the area of the mildewed rice areas.

## 3 RESULTS

### 3.1 Changes in box loss during model training

**FIGURE 3:**
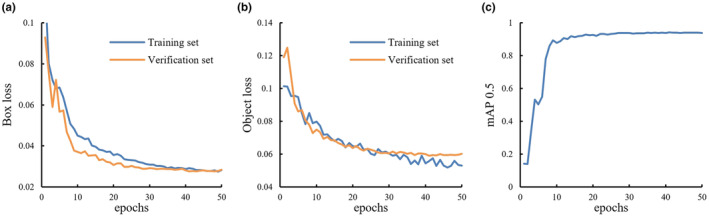
Parameter variation during model training. (a) Box loss (b) object loss (c) mAP 0.5.

Figure 3 shows the changes in box loss, object loss, and mAP_0.5_ during model training. As can be seen from the figure, during the initial stage of training, the box value in the training set and the verification set decreased rapidly. After 30 epochs, the loss value of the validation set stabilized. The mAP_0.5_ also stabilized after 30 epochs and was above 0.9.

### 3.2 Confusion matrix for the identification results of the rice mildew area detection model

The confusion matrix of detection results of the rice mold spot area detection model is shown in Table 1. In the training set and the verification set, the Precision with which the mildew areas were detected reached 89.3% and 86.5%, respectively, while the Recall of the mildew areas was 90.5% and 86.5%.

**TABLE 1:** YOLO model confusion matrix.

	Mildewed area (×10^3^ pixels)	Background area (×10^3^ pixels)	Precision (%)	Recall (%)
Training set				
Mildewed area	11,480	1377	89.3	90.5
Background area	1202	1,814,165		
Verification set				
Mildewed area	7320	1597	82.1	86.5
Background area	1143	1,209,101		

**FIGURE 4:**
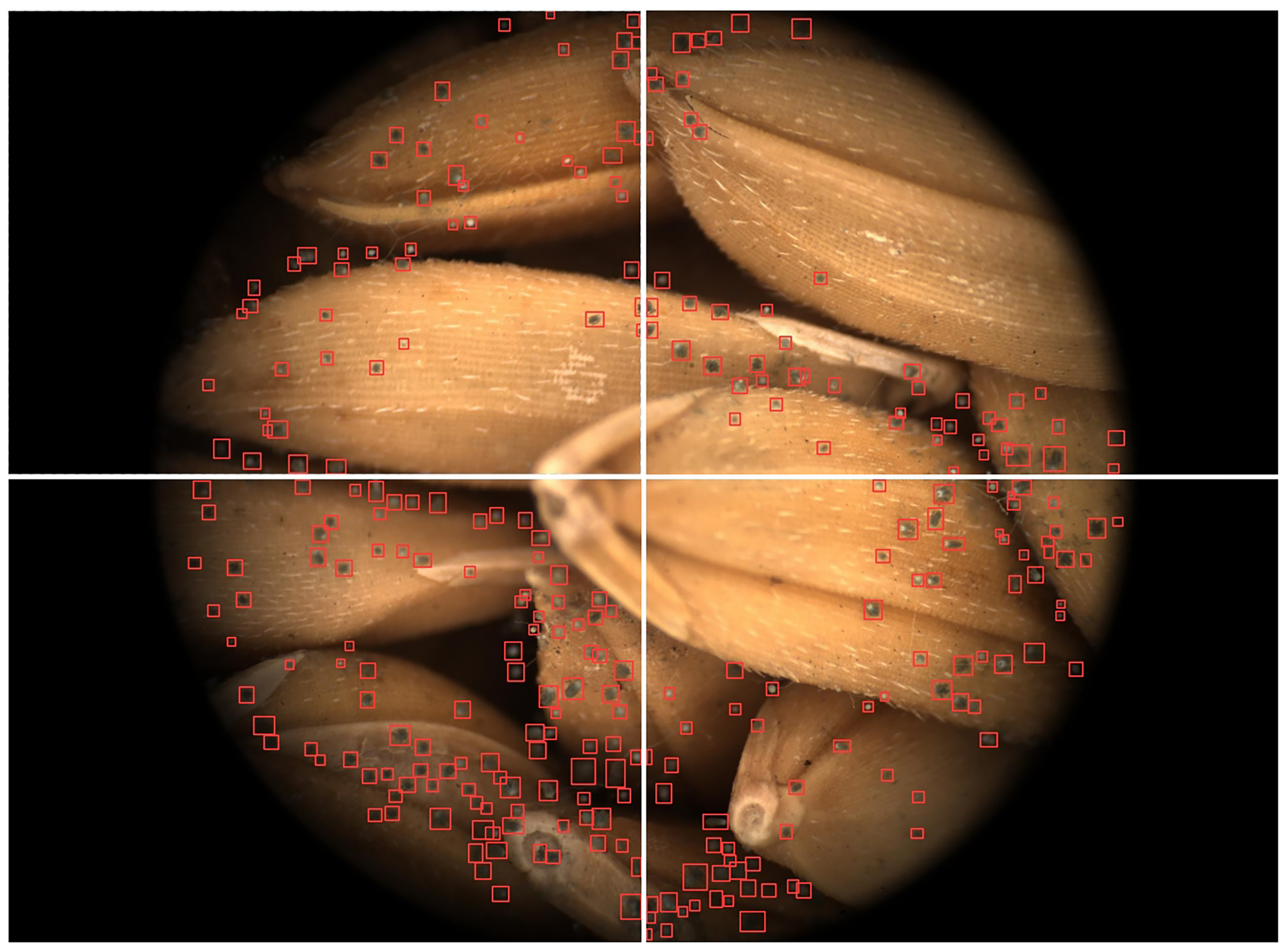
Mildewed areas detected by the YOLO‐v5 model in a rice MCV image.

Figure 4 shows an MCV image of the rice mold detected by the model, which was taken when the grain was mildly moldy (TVC within the range of 10^5^–10^6^ CFU/g). As can be seen from the figure, the proposed model can identify all mildewed areas in the image effectively, independent of the number of rice grains and the type of mold. In addition, the recognition speed of this model is very fast, and the time to analyze a single image was about 0.04 s.

### 3.3 Fitting degree of the bacterial count curve of unhulled and brown rice

**FIGURE 5:**
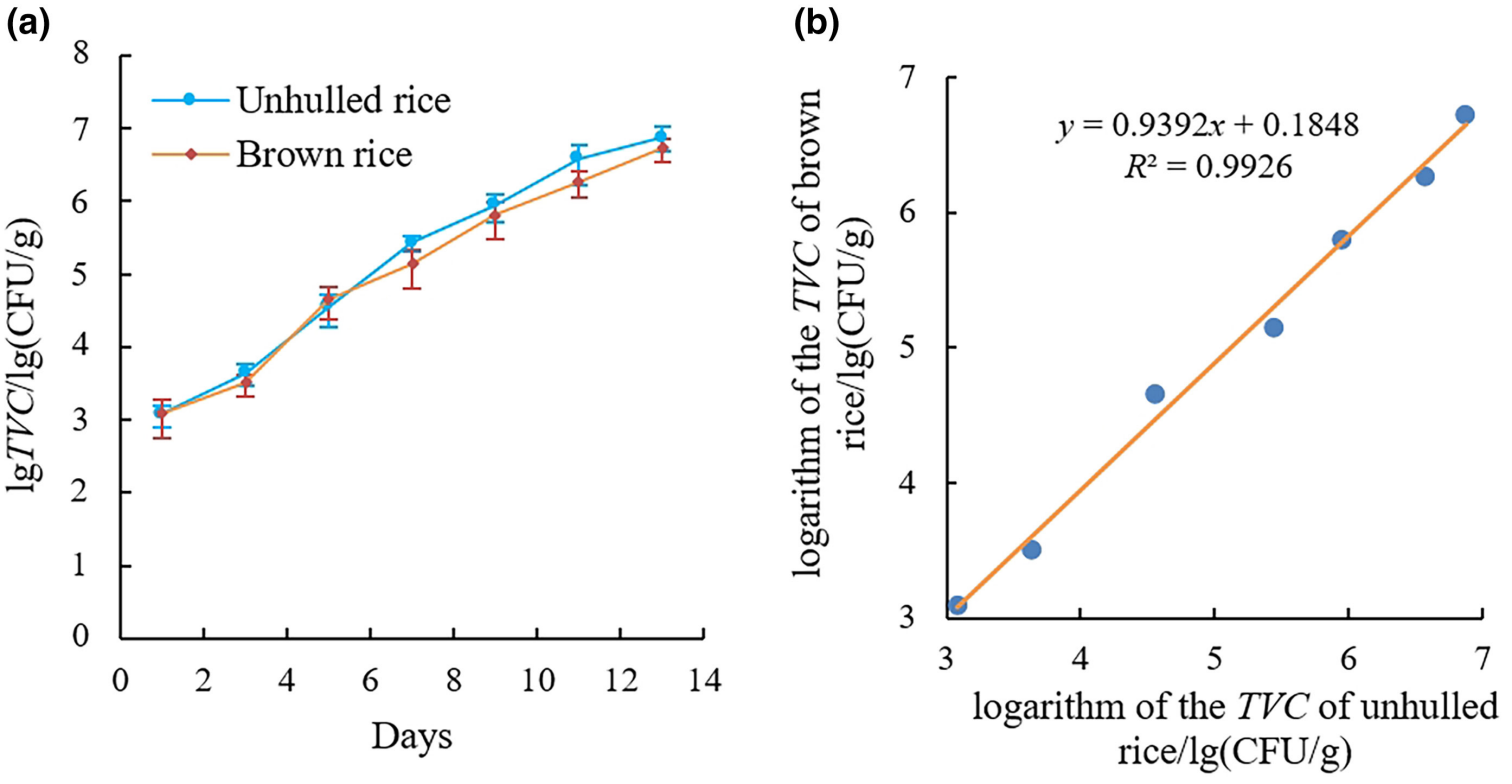
Changes in TVC of unhulled and brown rice. (a) TVC variation. (b) Relation between TVC of unhulled rice and brown rice.

Figure 5 shows the change in the TVC of unhulled and brown rice. With the increase in simulated storage days, the TVC of both rice types showed an exponential upward trend, and the mold growth rate of unhulled rice was faster than that of brown rice. On the 9th day of simulated storage, the TVC value was within the range of 10^5^–10^6^ CFU/g, and the grain was in a slightly moldy state; on the 11th day, the TVC value was greater than 10^6^ CFU/g, and the grain was in a severely moldy state. After correlation analysis, the determination coefficient *R* ^2^ of the logarithm of the TVC values of the unhulled rice and brown rice was 0.9926. The root mean square error (RMSE) of predicting the TVC value of brown rice using the TVC value of unhulled rice was 0.2025.

### 3.4 Analysis of the relationship between rice TVC and the area of mildewed areas identified by the model

**FIGURE 6:**
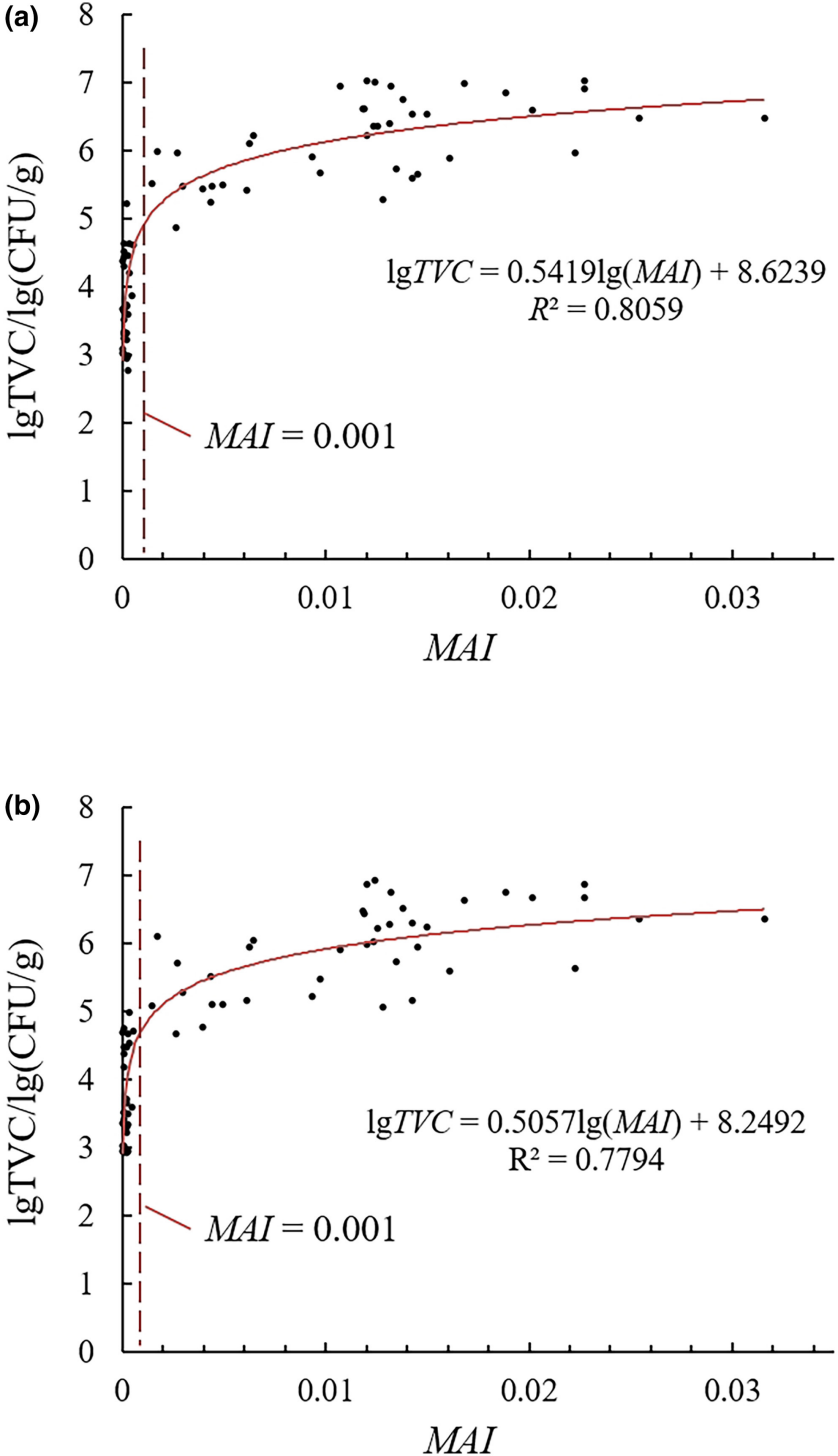
Relationship between the TVC of unhulled rice (a) and brown rice (b) and the area of mildewed areas identified by the model.

Figure 6 shows the relationship between the TVC of unhulled and brown rice and the area of the moldy rice area determined by the model (denoted using the letter *S*). According to the trend line equations, the area of mildewed areas identified by the model was linearly correlated with the logarithm of TVC for unhulled rice and brown rice. The *R* ^2^ of the regression models was 0.8059 and 0.7794, respectively. Specifically, the MAI values in 29 samples without significant mold (TVC < 10^5^ CFU/g, detected using brown rice) were lower than 0.001, while only 2 samples with light mold had MAI values above 0.001. The MAI values in 41 samples with light mold (10^5^ ≤ TVC < 10^6^ CFU/g, detected with brown rice) were all higher than 0.001. It can be seen that there is a clear threshold value for MAI for both unhulled and brown rice (MAI = 0.001) that can be used to distinguish between rice with no obvious mold and rice with a relatively light degree of mildew. Using this threshold MAI value, the Precision and Recall for light mold detection were 100% (41/41) and 95.3% (41/43).

## 4 DISCUSSION

The role of the YOLO model in this study was essentially to perform image segmentation, i.e. to divide the image into mildewed areas and background areas. However, mildewed areas in images are generally continuous and have complex shapes. The target boxes identified by the model or labeled via manual work rarely coincide completely with the real mildewed area. In order to formulate a model with higher precision and recall for mildewed area segmentation, CNN models for accurate image segmentation such as U‐net or Deeplab net can be used (Srinitya et al., 2023), but the object area labeling for these models is more complex than that of the YOLO model.

In recent years, scientific research on the identification and detection of moldy grains using electronic noses, hyperspectral imaging, and near‐infrared spectroscopy is continuously emerging. Jiarpinijnun et al. (2020) used an electronic nose combined with GC–MS analysis technology to detect characteristic volatile odors resulting from fungal contamination on brown rice grain, but this technology requires skilled operators to operate, and the detection process is costly and time‐consuming, and consequently not suitable for rapid on‐site detection. Jia et al. (2022) used hyperspectral technology to detect moldy corn seeds. Hyperspectral cameras are costly, and extracting hyperspectral image data requires significant data processing capabilities from operators. Therefore, this method is also not suitable for the practical production testing process. In addition, the above two methods can only detect moldy grains but cannot distinguish between different moldiness degrees. In this study, by analyzing the relationship between the TVC value of rice and the area of the mildewed areas identified by the model, it is possible to effectively distinguish between non‐significantly moldy rice and slightly moldy rice. Liu et al. (2023) used near‐infrared spectroscopy to perform the classification of sunflower seed mold grades. Although this technology can detect slight mold with some accuracy, the spatial resolution of near‐infrared spectroscopy is very low. If this technology is applied to rice detection, changes in the content of the fatty acids in rice will cause changes in their near‐infrared spectroscopic characteristics, which will increase the detection error (Cong et al., 2018). The MCV technology used in this study has low cost and good real‐time detection performance. Compared with traditional computer vision (Sun et al., 2016), the obtained microscopic images have higher resolution and capture detailed information from the rice samples, thereby achieving higher detection accuracy for moldy rice.

Compared with the study by Sun et al. (2022), the detection target for the experiment in this study was 10 g of rice rather than single grains of rice. By identifying and analyzing the mildewed areas of grouped samples, the obtained results can play a more effective role in practical applications of mold detection in large batches of rice. In this study, a bacterial suspension prepared by eluting spores and colonies was inoculated on the surface of naturally moldy rice, which resulted in more and richer types of molds. While MCV image capture will inevitably lead to a reduction in the visual field of the sample, in this study, a 9‐point acquisition method is adopted to collect microscopic images of grouped rice grains and to comprehensively process and analyze 9 images of the same group of rice samples. Thus, the obtained image data are more representative. Due to the fact that the current detection target for mildew in rice is based on the brown rice portion inside the rice husk, in this study, the degree of fitting of the TVC values of unhulled and brown rice was analyzed, and it was found that the determination coefficient *R* ^2^ between the two values was close to 1. The RMSE value of predicting the TVC value of brown rice using the TVC value of unhulled rice was relatively small, and the degree of mildew in unhulled rice follows a similar growth pattern to that encountered in brown rice for the same incubation time. Therefore, the degree of mildew in unhulled rice can be used to estimate the degree of mildew in brown rice, thus eliminating the tedious steps of hulling during rice mildew detection, which makes the detection process faster and more efficient. Moreover, in this study, the types of molds that contaminated the rice were not clearly distinguished. Regardless of whether it is unhulled or brown rice, the thresholds obtained clearly distinguish between no significant mold and mild mold change, so the experimental results will not be affected by different mold diffusion capabilities and visibility differences.

Through this study, it was found that the established Yolov5 model can be used to detect mildewed areas in grouped samples of rice effectively, and the method is capable of detecting even slightly moldy rice.

## 5 CONCLUSION AND PROSPECT

In this article, a YOLO‐v5 model is established to automatically detect mildewed areas in MCV images of rice mildly contaminated by mold. Using this model, mildewed unhulled rice samples with a TVC of 10^5^–10^6^ CFU/g can be detected using the MAI threshold. Compared with traditional methods, this method is real‐time and low‐cost, and can be widely used in rice safety evaluation in the rice market. In the future, this method should be tested using rice samples collected in grain depots or markets to verify its practicality. Moreover, in order to obtain a model with higher precision and recall, the segmentation effect of U‐net or Deeplab net for mildewed area of rice in MCV image can be tested.

## AUTHOR CONTRIBUTIONS

**Ke Sun:** Conceptualization (equal); data curation (equal); funding acquisition (equal); methodology (equal); software (equal). **Mengdi Tang:** Formal analysis (equal); investigation (equal); writing – original draft (equal). **Shu Li:** Investigation (equal); writing – review and editing (equal). **Siyuan Tong:** Investigation (equal); writing – review and editing (equal).

## FUNDING INFORMATION

This work was financially supported by Natural Science Foundation of Anhui Province, China: 2008085QC143 (Project leader: Ke Sun).

## CONFLICT OF INTEREST STATEMENT

All the authors declare that they have no conflict of interest.

## Data Availability

The data presented in this study can be found at Mendeley Data, V1, doi: 10.17632/cdjyh5ytp8.2.

## References

[fsn33798-bib-0001] Barsanti, L. , Birindelli, L. , & Gualtieri, P. (2022). Water monitoring by means of digital microscopy identification and classification of microalgae. Environmental Science‐Processes & Impacts, 23(10), 1443–1457. 10.1039/d1em00258a 34549767

[fsn33798-bib-1002] Cao, C. Y. , Zheng, J. C. , Huang, Y. Q. , Liu, J. , & Yang, C. F. (2019). Investigation of a promoted you only look once algorithm and its application in traffic flow monitoring. Applied Sciences‐Basel, 9(17), 3619.

[fsn33798-bib-0002] Chen, S. , Xiong, J. , Guo, W. , Bu, R. , Zheng, Z. , Chen, Y. , Yang, Z. , & Lin, R. (2019). Colored rice quality inspection system using machine vision. Journal of Cereal Science, 88, 87–95.

[fsn33798-bib-0003] Chen, W. , Huang, H. , Peng, S. , Zhou, C. , & Zhang, C. (2021). YOLO‐face: a real‐time face detector. Visual Computer, 37(4), 805–813.

[fsn33798-bib-0004] Cong, S. , Sun, J. , Mao, H. , & Wu, X. , Wang, P. , Zhang, X. (2018). Non‐destructive detection for mold colonies in rice based on hyperspectra and GWO‐SVR. Journal of the Science of Food and Agriculture, 98(4), 1453–1459. 10.1002/jsfa.8613 28786119

[fsn33798-bib-0005] Gong, Y. H. , Yang, T. J. , Liang, Y. T. , Ge, H. Y. , & Shen, E. B. (2021). Comparative assessments between conventional and promising technologies for wheat aging or mold detection. Cereal Research Communications, 49(4), 511–519.

[fsn33798-bib-0006] Jia, Y. , Li, Z. , Gao, R. , Zhang, X. , Zhang, H. , & Su, Z. (2022). Mildew recognition on maize seed by use of hyperspectral technology. Spectroscopy Letters, 55(4), 240–249.

[fsn33798-bib-0007] Jiang, X. S. , Zhao, T. X. , Liu, X. , Zhou, Y. C. , & Zhou, H. P. (2018). Study on method for on‐line identification of wheat mildew by array fiber spectrometer. Spectroscopy and Spectral Analysis, 38(12), 3729–3735.

[fsn33798-bib-0008] Jiang, Z. , Liu, X. , Yan, Z. , Gu, W. , & Jiang, J. (2021). Improved detection performance in blood cell count by an attention‐guided deep learning method. OSA Continuum, 4(2), 323–333.

[fsn33798-bib-0009] Jiarpinijnun, A. , Osako, K. , & Siripatrawan, U. (2020). Visualization of volatomic profiles for early detection of fungal infection on storage jasmine brown rice using electronic nose coupled with chemometrics. Measurement, 157, 107561.

[fsn33798-bib-0010] Li, S. , Feng, Z. , Yang, B. , Li, H. , Liao, F. , Gao, Y. , Liu, S. , Tang, J. , & Yao, Q. (2022). An intelligent monitoring system of diseases and pests on rice canopy. Frontiers in Plant Science, 13, 972286. 36035691 10.3389/fpls.2022.972286 PMC9403268

[fsn33798-bib-0011] Liu, J. , Fan, S. , Cheng, W. , Yang, Y. , Li, X. , Wang, Q. , Liu, B. , Xu, Z. , & Wu, Y. (2023). Non‐destructive discrimination of sunflower seeds with different internal mildew grades by fusion of near‐infrared diffuse reflectance and transmittance spectra combined with 1D‐CNN. Food, 12(2), 295. 10.3390/foods12020295 PMC9858067 36673386

[fsn33798-bib-0012] Mannaa, M. , Oh, J. Y. , & Kim, K. D. (2017). Biocontrol activity of volatile‐producing *Bacillus megaterium* and *Pseudomonas protegens* against *Aspergillus flavus* and *aflatoxin* production on stored rice grains. Mycobiology, 45(3), 213–219. 29138628 10.5941/MYCO.2017.45.3.213 PMC5673519

[fsn33798-bib-0013] Pan, L. , Wang, Z. , Sun, K. , Jia, X. , Du, L. , Yuan, J. , & Tu, K. (2017). Detection of paddy mildew degree based on computer vision. Transactions of the Chinese Society of Agricultural Engineering, 33(3), 272–280.

[fsn33798-bib-0014] Redmon, J. , Divvala, S. , Girshick, R. , & Farhadi, A. (2016). You Only Look Once: Unified, real‐time object detection. In Proceedings of the 2016 IEEE Conference on Computer Vision and Pattern Recognition (CVPR), Las Vegas, NV, USA, 27–30 June 2016; pp. 779–788.

[fsn33798-bib-0015] Santos, A. S. , Carreiro, F. , Freitas, A. , Barros, S. , Brites, C. , Ramos, F. , & Silva, A. S. (2022). Mycotoxins contamination in rice: Analytical methods, occurrence and detoxification strategies. Toxins, 14(9), 647. 36136585 10.3390/toxins14090647 PMC9504649

[fsn33798-bib-0016] Shen, F. , Wu, Q. , Wei, Y. , Du, L. , & Tang, P. (2016). Electronic nose and GC‐MS detection of volatile substances produced by mould strains. Journal of the Chinese Cereals and Oils Association, 31(7), 148–152. +156.

[fsn33798-bib-0017] Srinitya, G. , Sharmila, D. , Logeswari, S. , & Raja, S. D. M. (2023). Automated SAR image segmentation and classification using modified deep learning. International Journal of Pattern Recognition and Artificial Intelligence, 37(1), 2252027.

[fsn33798-bib-0018] Sun, K. , Wang, Z. , Tu, K. , Wang, S. , & Pan, L. (2016). Recognition of mould colony on unhulled paddy based on computer vision using conventional machine‐learning and deep learning techniques. Scientific Reports, 6, 37994. 27897236 10.1038/srep37994 PMC5126562

[fsn33798-bib-0019] Sun, K. , Zhang, Y. J. , Tong, S. Y. , Tang, M. D. , & Wang, C. B. (2022). Study on rice grain mildewed region recognition based on microscopic computer vision and YOLO‐v5 model. Food, 11(24), 4031. 10.3390/foods11244031 PMC9777938 36553773

[fsn33798-bib-0020] Wan, L. H. , Qu, C. L. , Xue, F. , & Wang, R. L. (2019). Study on the relationship between the degree of paddy mildew and the changes of its sensitive quality. Cereals & Oils, 32(9), 71–75.

[fsn33798-bib-0021] Wang, J. , Jiang, H. , & Chen, Q. (2021). High‐precision recognition of wheat mildew degree based on colorimetric sensor technique combined with multivariate analysis. Microchemical Journal, 168, 106468.

[fsn33798-bib-0022] Wen, F. R. , Guan, H. O. , Ma, X. D. , Zuo, F. , & Qian, L. (2022). Moldy rice detection method based on near infrared spectroscopy image processing technology. Spectroscopy and Spectral Analysis, 42(2), 428–433.

[fsn33798-bib-0023] Zhou, J. , Ju, X. , Sun, X. , Jin, H. , Yao, M. , & Shen, H. (2008). Succession of mould flora for paddy in different storage conditions. Journal of the Chinese Cereals and Oils Association, 5, 133–136.

